# Monthly Summary

**Published:** 1871-11

**Authors:** 


### MONTHLY SUMMARY.

*Treatment of Headache.*—Dr. C. J. Cleborne says in the *Medical Record:* Of course, in the treatment of this affection, whether it be idiopathic or symptomatic, the most important indication is to discover and remove the cause. But among the various remedies used for its relief, it would seem that the application of heat and cold has not received the attention it deserves. Now. instead of applying to the *head* a cloth wrung out in cold water, or an evaporating lotion, a more rapid and much better effect can be produced by the same applications applied to the *neck;* or a piece of intestine of the required length may be filled with ice water or pounded ice, and wrapped like a collar round the same part. Should it be necessary to use cold water to the head, a "beef-bladder" filled with ice water or ice (the neck of the bladder being tied around a cork) is a most efficient and clean mode of employing it. Heat may be applied in a similar manner, and will often succeed when cold fails to produce relief. The hot water may be put into a bladder, or into a rubber bag closed by a clamp, which may be obtained at a moderate cost. As a counter-irritant, I prefer a plaster made of freshly powdered capsicum to that of mustard, as it is quite as efficient, more cleanly, and may be left on for a number of hours withont blistering the tenderest skin. In a case of severe nervous headache the happiest effect may sometimes be obtained from a capsicum plaster placed over the nape of the neck, shifting it when necessary, or when it becomes unbearable, to the forehead or between the scapula, at the same time constantly applying to the face sponges squeezed out in *very hot* water. By this means the headache has often been quickly relieved, the patient falling asleep, and waking free from pain. When the headache is accompanied by throbbing in the carotids, temples, or forehead, a sensation of fulness, pressure, or heaviness over the eyes, a "pumping headache," as it is sometimes called, prompt relief may sometimes be obtained by cold applications to neck, and a capsicum plaster or hot application to epigastrium. If there be much nervous excitement, with a quick irritable pulse, small doses of tincture of digitalis or of aconite will be of service. For the irregularity of pulse and other symptoms accompaning a throbbing or "pumping headache," especially if there be giddiness, flushing of the face, etc., one-twelfth or one-sixteenth gr. doses of extract of belladonna, every two or four hours, will be found invaluable. For persons of studious, sedentary, or intemperate habits, headache, particularly the variety known as *Clavus*, will generally yield to the acetate of strychnia, extract of nux vomica, or extract of ignatia amara. In nearly all cases the judicious employment of cold or heat will greatly facilitate the treatment, and of themselves, often suffice to cure.—*Medical and Surgical Reporter.*

---

*Treatment of Poisoning by Carbolic Acid.*—Mr. Charles Roberts remarks that the indications for treatment are to remove the poison from the stomach as speedily as possible, to neutralize its action, and to treat the general symptoms of collapse in the ordinary way. A mixture of olive oil and castor oil has been recommended, and employed in some cases, with the object of diluting and carrying off the poison by the bowels, on the theory that it acts only as a corrosive, and is not absorbed. As we know that it is absorbed, it would be doubtful practice to continue this treatment and to make the acid run the guantlet of the fat absorbing surfaces of the small intestines. As carbolic acid is very slightly soluble in water, probably the speediest and most effectual way of removing it mechanically from the stomach would be to administer large quantities of warm water, or of mustard and water. As it is very soluble in glycerine, that substance with water and sulphate of zinc might be employed after the bulk of the poison had been removed by the former plan. From the serious action of the acid on the mucous membrane, the stomach pump should be employed with great care, and probably would often be inadmissible. Mr. Roberts states that he knows of no substance capable of neutralizing the acid chemically, but its well-known affinity for albuminous compounds would point to eggs and finely mixed or powdered *raw* meat as likely to prove of service. If eggs were used, it would be necessary, for obvious reasons, that they should be very much diluted by being whipped up with milk or cold water. Milk is not coagulated by carbolic acid, and therefore would not act as a neutralizer, but it would be a more suitable application than oil to the injured mucous membrane, and less likely to produce further discomfort to the patient. The general symptoms of collapse must be treated in the usual manner by internal stimulants, and friction and warmth to the skin. The rectum would be the most suitable part to which stimulants should be applied. If raw meat were given, it might be well seasoned, As brandy dissolves carbolic acid, and is itself speedily absorbed. its administration by the stomach would be contra-indicated.—*British Medical Journal.*

---

*Bone Felon.*—Of all painful things can there be any so excruciatingly painful as bone felon? We know of none that flesh is heir to. As this malady is quite frequent and subject of much earnest consideration, we give the latest recipe for its cure, which is given by that high authority, the London *Lancet:*

"As soon as the disease is felt put directly over the spot a fly blister, about the size of your thumb nail, and let it remain for six hours, at the expiration of which time, directly under the surface of the blister may be seen the felon, which can be instantly taken out with the point of a needle or a lancet."—*Druggists' Circular.*

---

*Salivation Consequent upon Artificial Teeth.*—Dr. Charles E. Buckingham in answer to Dr. O'Connell's question, in the Journal of June 29th, says that "I have seen several cases such as he describes, where the teeth were upon red rubber plate. At this moment I can recall three such cases seen with a twelvemonth."—*Boston Med. and Surg. Journal.*

*Citrate of Caffein in Neuralgia.*—Dr. G. W. Arnett, of Bossier Parish, Louisiana, reports a number of cases in which he has had great success in the treatment of neuralgia by the use of citrate of caffein and sulphate of morphia; also in nervous headache, hysteria, and other similar diseases. His prescription varies in amount to suit the case, the average being—

Sulph. morphia, gr. ss.;
Caffein, grs. iij.;
Citric acid, gr. iij.

to be given in some warm coffee, or, what is better, in a decoction of race ginger. The caffein and citric acid will, in the majority of cases, relieve nervous irritation without the addition of the morphia, which is a desideratum when the bowels are constipated. Its acts powerfully on the skin, equalizes the circulation, and thereby removes local congestion.—*Georgia Medical Companion.*

---

*Pyroxyline Dissolved in Oils.*—*Xylonite* differs from *Parkesine* in respect of the solvents employed—fixed oils, such as castor and linseed oils, being used for this purpose, as well as wood naphtha, alcohol, and other of the hitherto well-known solvents. In order to render the oils solvents of pyroxyline, it is necessary to heat them previously, then dissolve a portion of camphor in them, after which they become solvents of pyroxyline. This, Mr. Spiller pointed out, is a new fact in science. The cotton used for this purpose was the lowest form of gun cotton, and burnt very slowly on account of its low nitration. The temperature of the oil has to be raised to 300° Fah. in order to dissolve the cotton. The cotton is prepared by immersing it in four parts of sulphuric acid to one part of nitric acid at a low temperature. —*British Journal of Photography.*

---

*Advantages of Methylene as an Anæsthetic.*—Dr. Rendle, of Guy's Hospital, writes to the *Lancet* in favor of the use of methylene. He considers its advantages to be that, when properly administered, it is only exceptionally followed by nausea or sickness, both of which have followed the use of nitrous oxide gas. Its portability as compared with the latter is also an advantage. He states he has given it for operations lasting one hour, when the operator was able to commence in three minutes and recovery was rapid and not followed by sickness; also for operations lasting less than a minute, when all was finished and the patient sitting up within five minutes without the slightest unpleasant sensation.—*Lancet.*

---

### EDITORIAL DEPARTMENT.

